# Enhanced Recovery after Renal Transplantation Decreases Recipients’ Urological Complications and Hospital Stay: A Systematic Review and Meta-Analysis

**DOI:** 10.3390/jcm10112286

**Published:** 2021-05-25

**Authors:** Apostolos Prionas, Charles Craddock, Vassilios Papalois

**Affiliations:** 1 Department of Surgery and Cancer, Imperial College London, London SW7 2AZ, UK; v.papalois@imperial.ac.uk; 2 Department of General Surgery, Barking, Havering and Redbridge University Hospitals NHS Trust, London RM7 0AG, UK; charlescraddock@gmail.com

**Keywords:** solid organ transplantation, enhanced recovery after surgery, kidney transplantation, postoperative complications, length of hospital stay, urological complications, readmissions

## Abstract

The objective of this study was to compare enhanced recovery after surgery (ERAS) against traditional perioperative care for renal transplant recipients. Outcome measures included complications, length of stay (LOS), readmission rates, graft and patient survival up to one-year post-transplant. We initially screened Medline, Cochrane, Scopus, Embase and Web of Science databases. We identified 3029 records. From these, 114 full texts were scrutinized for inclusion. Finally, 10 studies were included in the meta-analysis corresponding to 2037 renal transplant recipients. ERAS resulted in lower incidence of urological complications (95CI: 0.276, 0.855) (I^2^ = 53.08%) compared to traditional perioperative practice. This referred to ureteric stenoses (95CI: 0.186–0.868) (I^2^ = 0%) and urinary tract infections (95CI: 0.230–0.978) (I^2^ = 71.55%). ERAS decreased recipients’ LOS (95CI: −2.876, −0.835) (I^2^ = 86.55%). Compared to standard practice, ERAS protocols did not increase unplanned readmissions (95CI:0.800, 1.680) (I^2^ = 0%). Up to one-year post-transplant, graft survival rates were similar across the ERAS and the control groups (95CI:0.420, 1.722) (I^2^ = 0%). There was also no difference in recipients’ one-year post-transplant survival (95CI:0.162, 3.586) (I^2^ = 0%). Our results suggest that ERAS protocols can be safely incorporated in the perioperative care of renal transplant recipients, decrease their urological complications and shorten their length of hospital stay without affecting unplanned readmission rates.

## 1. Introduction

Renal transplantation is the optimal treatment for individuals with end-stage renal disease (ESRD) [1]. The associated improvements in quality of life, mortality, morbidity and cost savings are substantial in comparison to dialysis [1]. Despite this, the rates of renal transplantation remain considerably low. For example, in Europe in 2019 only 36 renal transplants per million population (pmp) were performed, whereas 854 patients pmp were on dialysis therapy [2].

As transplant patients are frailer, undergo more complex procedures, experience greater peri-operative morbidity and have longer admissions compared to the majority of surgical patients, limited resource availability has become a major obstacle partly due to persistently high bed occupancy at renal transplant centers [3]. In order to widen access to transplantation, it is therefore important to ensure that clinical pathways are developed that optimize both the utilization of resources, as well as the safety and efficacy of transplantation surgery.

Enhanced recovery after surgery (ERAS), a principle initially applied to colorectal surgery, aims to minimize peri-operative physiologic dysfunction and the surgical stress response in order to promote a swifter return to normal function [4]. As such, ERAS depicts a major alteration in the organization of the perioperative care and incorporates a multimodal approach to deliver a powerful synergistic impact [4].

Our meta-analysis aims to compare ERAS against standard care for renal transplant recipients. Moreover, we sought to identify the existing ERAS interventions pre-operatively, intra-operatively and post-operatively and synthesize a cutting-edge ERAS protocol for renal transplantation.

## 2. Materials and Methods

### 2.1. Protocol 

This meta-analysis is part of a broader academic project. Another study (part of the same project) that demonstrates the safety and the efficacy of ERAS in the perioperative care of living kidney donors has already been published by our team earlier this year. The project’s protocol is registered in the Imperial College’s database. 

### 2.2. Eligibility Criteria

In our review, we included:

- Randomized Clinical Trials (RCTs), retrospective and prospective cohort studies.
- Studies that included adults with ESRD undergoing renal transplantation.
- Studies including multiple ERAS interventions or streamlined protocols. Any intervention that aimed to minimize the peri-operative physiologic dysfunction and the surgical stress response in order to promote a swifter return to normal function was considered as an ERAS intervention. For a study to meet our eligibility criteria, two or more synergistic ERAS interventions were required.
- Studies where traditional perioperative practice was the main comparator.
- Studies reporting complications, grafts’ and patients’ survival, duration of hospitalization, unplanned readmissions and quality of life data in the intervention and the comparator groups.

We excluded:

- Studies in animals or cadavers, comments/letters to the editor, opinion papers, case studies, systematic reviews, meta-analyses, congress abstracts when an analytic report was not available and studies in which multiple synergistic ERAS interventions were not included.
- Studies enrolling pediatric patients or patients undergoing any different operation other than renal transplantation, as well as studies that excluded participants with significant post-operative complications.
- Studies involving a single intervention. Our meta-analysis seeks to investigate the synergistic effect of different ERAS interventions (care bundles) in the perioperative care of kidney transplant recipients. Thus, articles on solitary interventions were not included.
- Non-comparative studies and articles in which the relevant outcome data were not reported.

### 2.3. Search Strategy 

We screened Medline, Cochrane, Scopus, Embase and Web of Science databases until 5 October 2020 using an advanced search strategy. To reduce publication bias, we included grey literature in our search strategy. We only considered detailed reports of unpublished studies to be eligible for inclusion in our systematic review.

### 2.4. Selection of Studies

We initially transferred the identified records into a reference management software (Endnote). We then eliminated the duplicate records. The remaining records were scrutinized by two authors who decided which full-texts should be sought and screened for inclusion. We finally assessed the quality of the articles that were included in our systematic review (size, follow up, bias etc.). 

### 2.5. Data Collection 

A specifically designed standard operation spreadsheet was used for data extraction. We did not communicate with any of the studies’ authors in order to obtain additional data.

### 2.6. Extracted Data 

We extracted data on:

- Recipients’ Baseline Characteristics: gender (number of male recipients, % percentage), age (in years, mean ± standard deviation or median, (range)), Body Mass Index (BMI in kg/m^2^), recipients’ smoking history, recipients’ comorbidities, hemodialysis (number of recipients receiving hemodialysis, % percentage), time in hemodialysis (in months), previous renal transplant (number of recipients who had previous renal transplant, % percentage), deceased donor kidney transplant (number of recipients receiving kidney from deceased donors, % percentage), diabetic nephropathy (number, % percentage), left kidney transplant (number, % percentage), ABO incompatibility with donor (number, % percentage), positive tissue crossmatch (number, % percentage), HLA mismatch (number, % percentage), graft’s warm ischemia time (in hours), cold ischemia time (in hours) and overall ischemia time (in hours).
- Enhanced Recovery and traditional practice interventions: preoperatively, intraoperatively and postoperatively (qualitative data).
- Recipients’ outcomes:
  - One-year post-transplant patient survival (number, % percentage).
  - Complications: overall postoperative complications, Clavien-Dindo I–II complications, Clavien-Dindo III–V complications, urological complications, urinary leakage, ureteral stenosis/obstruction, urinary tract infection (UTI), vascular complications, vascular anastomotic leak, renal artery stenosis, renal vein/renal artery thrombosis, symptomatic lymphocele, delayed graft function, graft rejection, electrolyte disorders, gastrointestinal complications, infections other than UTI, other complications (number, % percentage).
  - One-year post-transplant graft survival (number, % percentage).
  - Length of Stay (LOS) (in days, mean ± standard deviation or median, (range)).
  - Readmissions (number, % percentage).
  - Reasons for readmissions (qualitative data).
  - Adverse events (qualitative data).
  - Quality of recovery score [5]

### 2.7. Risk of Bias in Individual Studies

In individual studies, we assessed the risk of bias using targeted assessment proformas, namely the “Risk-Of-Bias In Non-Randomized Studies of Interventions” (ROBINS-I) and the “Revised Cochrane Risk of Bias tool for Randomized Trials” (RoB 2) [6,7]. 

### 2.8. Summary Measures

We compared dichotomous variables using Odds Ratio (OR) and continuous variables using the difference in means.

### 2.9. Synthesis of the Results

For all meta-analyses, 95% Confidence Interval (CI) was used. Results were presented using forest plots. Random-effects statistical models were used. The average difference in means (ERAS-traditional perioperative care) or odds ratio (ERAS vs. traditional perioperative practice) were calculated. To estimate heterogeneity, we obtained I^2^. We performed the meta-analyses using the OpenMetaAnalyst software.

### 2.10. Risk of Bias across Studies

We sought to reduce across-study bias. We screened published and unpublished literature in an effort to reduce publication bias. The risk of bias in every different domain was assessed for each individual study as well as across the different study types (non-randomized studies vs. Randomized Clinical Trials). 

### 2.11. Additional Analyses

On a protocol level, we expected some degree of heterogeneity. More specifically, we expected that the ERAS and the traditional perioperative practice interventions would vary between the different studies. Where we identified heterogeneity, we attempted to explore it with subgroup meta-analyses, when possible.

## 3. Results

### 3.1. Selection of Studies

**Figure 1:**
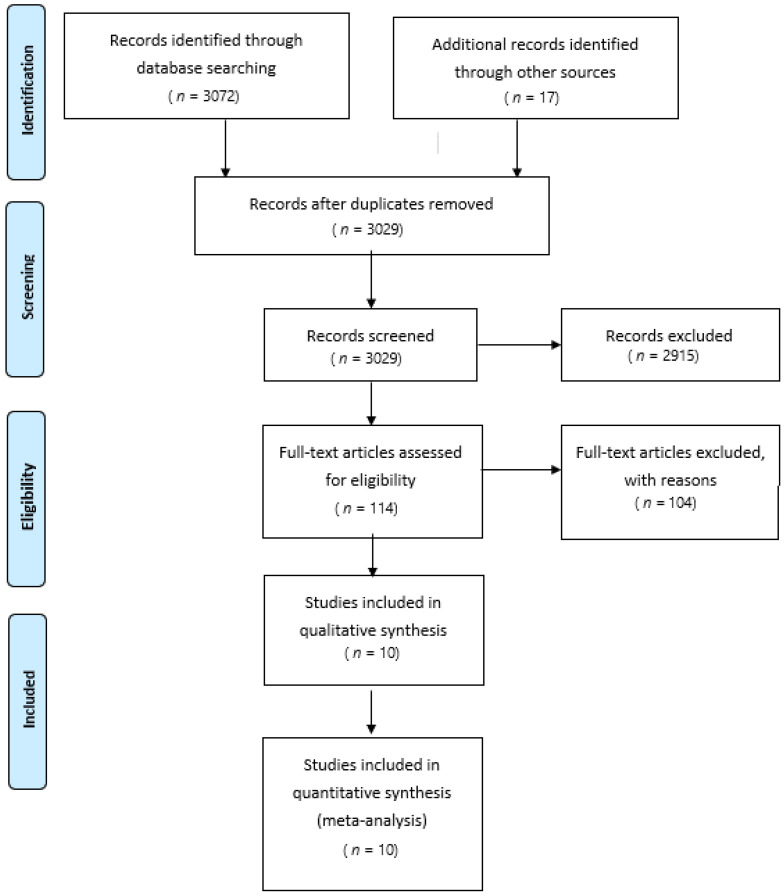
Systematic review flow diagram.

The systematic review’s flow chart is presented in Figure 1. The database search returned three thousand and seventy-two records. Seventeen records were identified through other sources (references of articles). Following elimination of duplicates, three thousand and twenty-nine abstracts were screened. Two thousand nine hundred and fifteen were excluded. One hundred and fourteen full-text publications were scrutinized. One hundred and four articles were excluded (eleven referring to ERAS protocols for living kidney donors; twenty-seven congress abstracts lacking the relevant data or presenting duplicated results; eight comments, reviews, opinion papers and protocols; three because they excluded participants with significant complications; three because they did not report relevant outcomes; one due to lack of a control group; three because they included pediatric participants; three referring to nephrectomy operations; forty-five because only a solitary intervention was reported). In total, ten studies, two randomized clinical trials and eight cohort studies were deemed eligible for inclusion in our systematic review and meta-analysis [8,9,10,11,12,13,14,15,16,17].

### 3.2. Study Characteristics and Bias Assessment

Table 1 and Table 2 summarize the qualitative assessments of the individual studies. Our systematic review refers to 2037 renal transplant recipients. The risk-of-bias assessment across the different study types can also be seen in Table 1 and Table 2. 

### 3.3. Included Studies’ Results

The characteristics of the renal transplant recipients are illustrated in Table 3 and Table 4. The different synergistic ERAS interventions included in the individual studies are summarized in Table 5. These ERAS interventions were compared with a variety of standard care interventions across the included studies. The most common standard care interventions were:

- Preoperative: fasting and intravenous maintenance fluids administration [8,9,10,11,12,13,14,15,16,17].
- Intraoperative: routine central venous catheter placement [10,11], liberal fluids administration [10,11], placement of externally draining percutaneous suprapubic stents [8,13] or double JJ stents that were removed after four weeks post-operatively [14,17].
- Post-operative: administration of opioid analgesics and no use of wound infiltration catheters and local anesthetic agents [8,9,10,11,12,13,14,15,16,17].

The recipients’ outcomes can be found in Table 6, Table 7 and Table 8. Only one study (Bruintjes, M. H. D. et al. [8]), assessed recipients’ quality of life data. The authors found that their ERAS recipients scored higher on the quality of recovery questionnaire in the first postoperative week compared to the standard care group. The studies included in the present systematic review did not capture any adverse events caused by ERAS interventions.

### 3.4. Synthesis of Results

#### 3.4.1. Qualitative Synthesis

The studies included in our systematic review and meta-analysis refer to an overall population of 2037 renal transplant recipients. Recipients were in the majority middle-aged men. The most common co-morbidities were diabetes mellitus, hypertension and ischemic heart disease. The vast majority of the recipients, either in the ERAS or the traditional perioperative care cohorts, were managed with hemodialysis for a period of approximately two years before the transplant. Approximately 1/10 recipients in each cohort had another renal transplant in the past. There were no significant differences in terms of demographics, BMI, co-morbidities, deceased donation rates, cold and overall ischemia times across the ERAS and the traditional perioperative practice groups. Different ERAS interventions were demonstrated across the included studies. These are summarized in Table 5. Among them, the placement of a double JJ stent intraoperatively and the removal of this stent in the early post-operative period (POD7-POD21) were considered the two single most effective interventions. 

#### 3.4.2. Quantitative Synthesis

##### Post-Operative Complications

**Figure 2:**
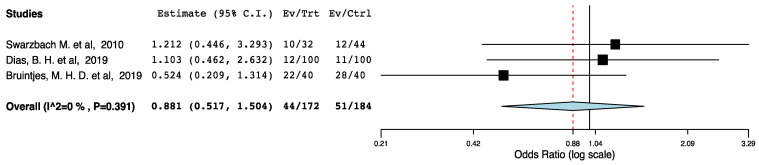
Forest plot: overall complications (ERAS vs. traditional perioperative care).

**Figure 3:**
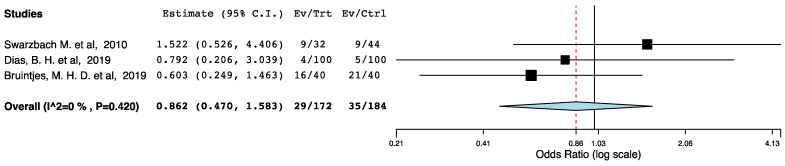
Forest plot: Clavien-Dindo I-II complications (ERAS vs. traditional perioperative care).

**Figure 4:**
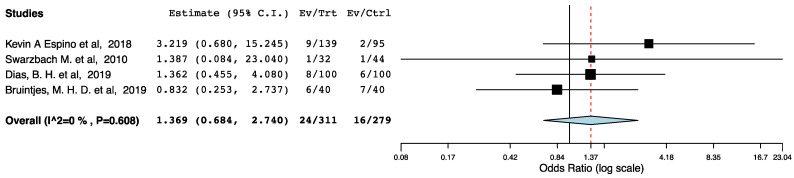
Forest plot: Clavien-Dindo III-V complications (ERAS vs. traditional perioperative care).

**Figure 5:**
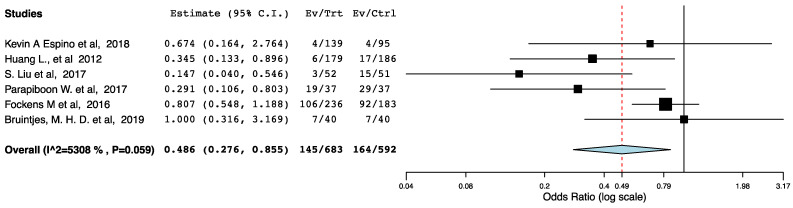
Forest plot: urological complications (ERAS vs. traditional perioperative care).

**Figure 6:**
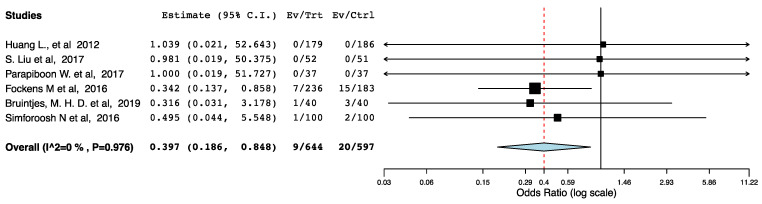
Forest plot: ureteric obstructions or stenoses (ERAS vs. traditional perioperative care).

**Figure 7:**
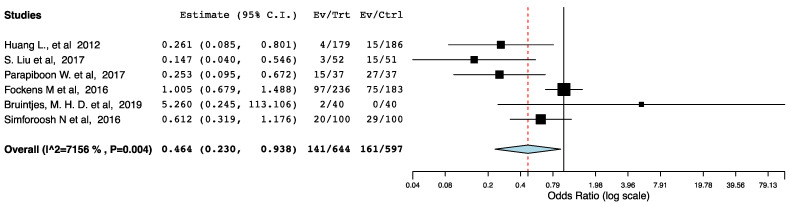
Forest plot: UTIs (ERAS vs. traditional perioperative care).

With regards to perioperative morbidity, we did not find a difference in the occurrence and the severity of post-operative complications across the ERAS and the control groups (Figure 2, Figure 3 and Figure 4). The ERAS recipients had less urological complications compared to the control groups (95CI: 0.276, 0.855) (*p* = 0.012) (I^2^ = 53.08%, Het *p* = 0.059) (Figure 5). This accounted for post-operative ureteric stenoses/obstructions and UTIs (Figure 6 and Figure 7). There was no difference on the incidence of urinary leaks and non-urological complications across the ERAS and the control groups. 

##### LOS

**Figure 8:**
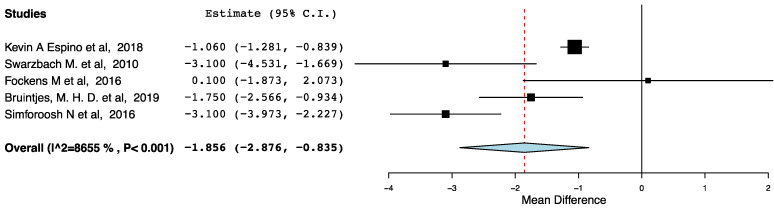
Forest plot: length of hospital stays (ERAS—traditional perioperative care). Only studies reporting LOS as mean ± SD where included in this meta-analysis.

Figure 8 shows that compared to patients receiving traditional perioperative care, recipients receiving ERAS spent less time in hospital (95CI: −2.876, −0.835) (*p* < 0.001) (I^2^ = 86.55%, Het *p* < 0.001). The observed statistical heterogeneity (I^2^ = 86.55%) was attributed to the difference in the effectiveness of the ERAS and standard care pathways in the included studies and could not be further explored.

##### Readmission Rates

**Figure 9:**
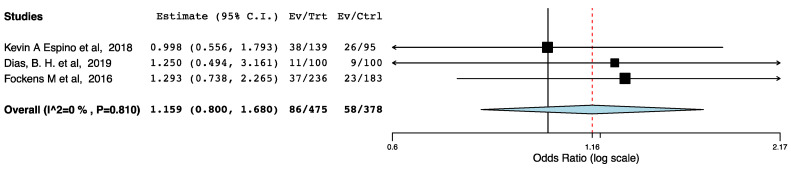
Forest plot: unplanned readmissions (ERAS—traditional perioperative care).

Three non-randomized studies provided data on recipients’ readmissions. As shown in Figure 9, the implementation of ERAS protocols in these studies did not result in increased readmissions rates (95CI: 0.800, 1.680) (*p* = 0.435) (I^2^ = 0%, Het *p* = 0.810).

##### One-Year Post-Transplant Graft Survival

**Figure 10:**
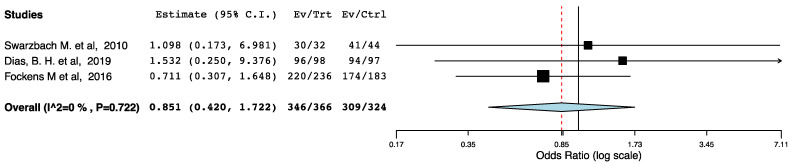
Forest Plot: One-year post-transplant graft Survival (ERAS vs. traditional perioperative care).

With regard to graft survival, three non-randomized studies provided follow-up data. Up to one year post-operatively, we did not find a difference in graft survival rates across the ERAS and the control groups (95CI: 0.420, 1.722) (*p* = 0.653) (I^2^ = 0%, Het *p* = 0.722) (Figure 10).

##### One-Year Post-Transplant Patient Survival

**Figure 11:**
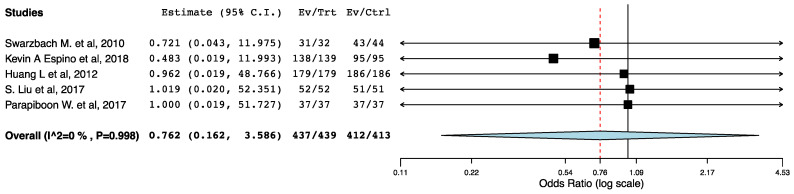
Forest plot: one-year patient survival (ERAS vs. traditional perioperative care).

Five studies, two RCTs and three non-randomized trials reported one-year post-transplant patient survival. We did not find a difference in recipients’ one-year survival across the ERAS and the control groups (95CI: 0.162, 3.586) (*p* = 0.731) (I^2^ = 0%, Het *p* = 0.998) (Figure 11).

##### Additional Analyses

**Figure 12:**
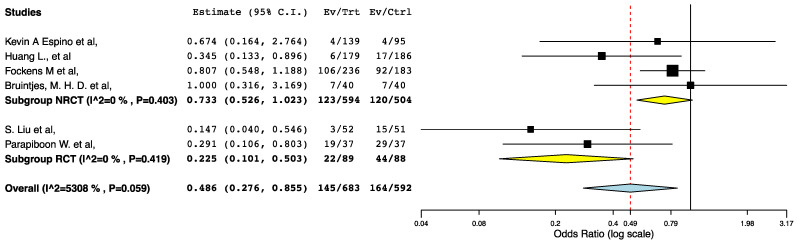
Forest plot: subgroup meta-analysis. (ERAS—traditional perioperative care).

We observed significant statistical heterogeneity in the meta-analysis for the incidence of urological complications. Being considerate of the fact that non-randomized data carry high risk of recall and selective reporting bias, we preformed subgroup meta-analyses of the randomized and non-randomized studies to explore the abovementioned heterogeneity. As illustrated in Figure 12, the subgroup meta-analysis of the RCTs included in the present systematic strongly supported that enhanced recovery after renal transplantation is associated with decreased incidence of urological complications (Level I evidence).

## 4. Discussion

### 4.1. Summary of Evidence

To our knowledge, this is the first systematic review and meta-analysis to compare ERAS against traditional practice for the perioperative care of renal transplant recipients. This meta-analysis showed that ERAS protocols can be safely incorporated in the management of renal transplant recipients. Most importantly, we showed that the implementation-enhanced recovery in this high-risk surgical population can decrease their urological complications and shorten their length of hospital stay without affecting unplanned readmission rates. Moreover, through reviewing the existing evidence corresponding to 2037 kidney transplant recipients, this study delivered a cutting-edge ERAS protocol for renal transplantation.

The primary aim of our review was to investigate whether ERAS can be safely incorporated in the perioperative care of renal transplant recipients. As illustrated in our systematic review, renal transplant recipients are usually frail individuals with ESRD, multiple co-morbidities and cardiovascular risk factors. Their safety is therefore of paramount importance. Enhanced recovery pathways primarily aim to mitigate surgical stress and expedite the recipients’ recovery. Our meta-analysis demonstrated that neither the recipients’ nor the grafts’ safety are compromised by ERAS. We did not find a difference in patient and graft survival rates across the ERAS and the control groups. We also did not capture any adverse events related to the ERAS protocols implemented in the included studies. Despite the fact that there was no difference on the overall (mild and severe) post-transplantation complications, our study revealed a clear signal for decrease in the occurrence of urological complications with ERAS. This primarily referred to ureteric stenoses and UTIs. These findings are particularly important. Despite the fact that similar evidence exists in other surgical elective fields, renal transplant recipients are possibly one the highest-risk populations where ERAS has been successfully implemented [18,19,20].

Furthermore, we sought to investigate the added benefits from the implementation of ERAS pathways following renal transplantation. Patients receiving ERAS had shorter LOS compared to standard care recipients. We did not find any difference on the incidence of unplanned readmissions after hospital discharge across the ERAS and the control groups. With regard to quality of life after renal transplantation, only one study reported recipients’ quality of life data [8]. The authors found that compared to traditional practice, ERAS resulted in better quality of recovery and pain control in the immediate post-operative period [8]. The implementation of enhanced recovery pathways in different surgical fields (e.g., colorectal surgery) has resulted in similar patients’ benefits [18,19,20].

Our secondary goal was to address the clinical need for a cutting-edge ERAS protocol for renal transplantation. We summarized the preoperative, intraoperative and post-operative ERAS interventions reported in the studies included our systematic review and streamlined them into a single ERAS renal transplantation protocol. This protocol followed the same principles (preoperative carbohydrate loading, placement of wound infiltration catheters, early mobilization and enteral nutrition postoperatively, etc.) as the living donors’ ERAS protocol published by our team earlier this year that was also shown to decrease patients’ length of stay and complications [21]. There were two important alterations though, namely the placement of a double JJ stent intraoperatively and the removal of this stent in the early post-operative period (POD7-POD21). The efficiency of these two interventions has been confirmed by multiple previous high-quality studies. Routine placement of JJ stents in renal transplantation has been found to decrease the incidence of major urological complication in the Cochrane systematic review by Wilson C. et al. [22]. Visser I et al. in their 2019 meta-analysis of randomized controlled trials found that early ureteric stent removal before the POD21 results reduced incidence of UTIs [23]. It is likely that these two synergistic interventions account for the greatest part of the reduction of the urological complications (ureter stenoses and UTIs) in our study population. Different theories have been suggested to explain how the placement of a JJ stent and its early removal can reduce the incidence of ureteric stenoses and UTIs [24,25,26]. The prophylactic placement of a JJ stent results in larger luminal diameter of the ureter post-operatively [24]. It provides mechanical support, as it prevents ureteral bending or collapse from external compression [24]. The stent also protects the anastomosis from the undesirable effects of increased intraluminal pressure, especially during the high-diuresis phase that patients commonly experience early post-transplantation; this increased pressure can potentially result in microscopic leaks, consequent fibrosis in the area and ureteric stenoses/obstruction [24]. If the JJ stent remains in place for long, it can become more easily colonized by urinary pathogens. Early removal of the stent seems to decrease the likelihood for the development of UTIs. Our results suggest that prophylactic double JJ stent placement and stent removal in the early post-operative period can minimize the peri-operative physiologic dysfunction and promote a swifter return to normal function following kidney transplantation. Thus, we consider these synergistic interventions as key features of our cutting-edge ERAS protocol for renal transplantation. 

### 4.2. Implications of Study Findings

Our meta-analysis showed that the implementation of enhanced recovery after renal transplantation can safely decrease the patients’ length of stay and urological complications with no undesirable increase in unplanned readmission rates. This is a major finding with multiple implications for ESRD patients and the transplantation system in general. Our findings suggest that ERAS has the potential to decrease patients’ peri-operative morbidity and therefore facilitate a swifter return to normal life. From a system’s perspective, decreasing the inpatient length of hospital stay is likely to lower bed occupancy rates in transplant centers and increase the availability of existing resources. Broad implementation of ERAS programs could therefore contribute towards increasing the existing low rates of renal transplantation.

### 4.3. Limitations

The present study had some limitations. Due to lack of reported data, some outcome parameters were only assessed by a minimum of three studies. There is risk of recall and selective reporting bias, evidenced by the inclusion of non-randomized data. Regarding consistency measures, we observed significant heterogeneity in the meta-analyses for two primary endpoints: LOS and urological complications. The source of heterogeneity in the meta-analysis for urological complications was further explored through a subgroup meta-analysis. The underlying etiology of the heterogeneity was identified and addressed. The subgroup meta-analysis of the RCTs included in the present systematic strongly supported that enhanced recovery after renal transplantation is associated with decreased incidence of urological complications. The heterogeneity in the LOS meta-analysis could not be further explored statistically. To our view, this heterogeneity is likely to originate from the difference in the effectiveness of the ERAS and standard care interventions in the included studies. The heterogeneity for the remaining meta-analyses was not significant. The risk of publication bias was considered to be low.

## 5. Conclusions

This study shows that ERAS protocols combining well-established generic ERAS interventions (preoperative carbohydrate loading, placement of wound infiltration catheters, early mobilization and enteral nutrition postoperatively, etc.) with prophylactic placement of double JJ stents and their early removal can be safely incorporated in the perioperative care of renal transplant recipients, decreasing their urological complications and shortening their length of hospital stay without affecting unplanned readmission rates. 

## Figures and Tables

**Table 1:** Non-randomized studies: quality and risk-of-bias assessment.

Authors, Year	Study Size (ERAS/Standard Care)	Length of Follow Up	Risk of Bias								
			Confounding	Selection of Participants	Classification of Interventions	Deviations from Intended Interventions	Missing Data	Measurement of Outcomes	Selection of the Reported Result	Overall	Direction of Bias
Bruintjes, M. H. D. et al., 2019 [8]	80 (40/40)	14 days	Serious	Serious	Low	Low	Low	Moderate	Moderate	Serious	Favors ERAS
Dias, B. H. et al., 2019 [9]	200 (100/100)	365 days	Serious	Low	Serious	Low	Low	Low	Low	Serious	Unpredictable
Kevin A Espino et al., 2018 [10]	234 (139/95)	90 days	Serious	Low	Low	Moderate	Low	Low	Low	Serious	Favors Standard Care
Halawa, A. et al., 2018 [11]	286 (135/151)	No info	Serious	Low	Serious	Low	Low	Low	Low	Serious	Unpredictable
Simforoosh N et al., 2016 [12]	200 (100/100)	365 days	Critical	No info	Serious	Low	Low	Low	Low	Critical	Unpredictable
Fockens M et al., 2016 [13]	419 (236/183)	365 days	Critical	Low	Low	Low	Moderate	Low	Low	Critical	Favors Standard Care
Huang L., et al. 2012 [14]	365 (179/186)	90 days	Low	Low	Low	Low	Low	Low	Low	Low	Towards null
Swarzbach M. et al., 2010 [15]	76 (32/44)	365 days	Serious	Low	Low	Low	Low	Low	Low	Serious	Unpredictable

**Table 2:** Randomized clinical trials: quality and risk-of-bias assessment.

Authors, Year	Study Size (ERAS/Standard Care)	Length of Follow Up	Risk of Bias							
			Randomization Process	Effect of Assignment to Intervention	Effect of Adhering to Intervention	Missing Outcome Data	Measurement of the Outcome	Selection of the Reported Result	Overall	Direction of Bias
S. Liu et al., 2017 [17]	103 (52/51)	90 days	Low	Low	Low	Some Concerns	Low	Low	Some Concerns	Towards null
Parapiboon W. et al., 2017 [16]	74 (37/37)	30 days	Low	Some Concerns	Some Concerns	Low	Low	Low	Some Concerns	Unpredictable

**Table 3:** Recipients’ background (A).

Authors, Year	Recipients Age		Male Recipients		Recipients’ BMI		Recipients’ Comorbidities		Diabetic Nephropathy	
	ERAS	Standard Care	ERAS	Standard Care	ERAS	Standard Care	ERAS	Standard Care	ERAS	Standard Care
Non-Randomized Studies										
Bruintjes, M. H. D. et al., 2019 [8]	46.4 ± 15.4	53.5 ± 13.0	25/40 (62.5%)	17/40 (42.5%)	25.2 ± 3.3	24.3 ± 3.6	ASA (1/2/3/4): 0/8/30/2, Diabetes: 5/40 (12.5%)	ASA (1/2/3/4): 1/11/28/0, Diabetes: 3/40 (7.5%)	-	-
Dias, B. H. et al., 2019 [9]	51.4± 14.2	53.1± 14.3	54/100 (54%)	64/100 (64%)	-	-	Diabetes: 32/100 (32%), Hypertension: 72(72%), Previous abdominal surgery: 7/100 (7%)	Diabetes: 31/100 (31%), Hypertension: 69/100 (69%), previous abdominal surgery 6/100 (6%)	-	-
Kevin A Espino et al., 2018 [10]	-	-	78/ 139 (56.1%)	58/95 (61.1%)	-	-	Diabetes: 58/139 (41.7%)	Diabetes: 30/95 (31.6%)	-	-
Halawa, A. et al., 2018 [11]	48	50	127/135 (94%)	148/151 (98%)	-	-	-	-	-	-
Fockens M et al., 2016 [12]	55.0± 13.3	54.2± 12.8	145/236 (61.4%)	111/183 (60.7%)	27.2 ± 5.4	25.5 ± 4.3	Diabetes: 49/236 (20.8%) Cardiac history: 29/236 (12.3%)	Diabetes: 48/183 (26.2%) Cardiac history: 31/183 (26.2%)	49/236 (40.8%)	23/183 (12.6%)
Simforoosh N et al., 2016 [13]	42 (18–58)	40 (19–60)	60/100 (60%)	36/100 (36%)	26.02 ± 5.87	24.2 ± 5.18	Diabetes: 72/100 (72%)	Diabetes: 73/100 (73%)	-	-
Huang L., et al. 2012 [14]	43.5 ± 8.1	42.8 ± 7.5	133/179 (74.3%)	137/186 (73.6%)	-	-	-	-	4/179 (2.2%)	5/186 (2.7%)
Swarzbach M. et al., 2010 [15]	55.5	48.9	20/32 (62.5%)	29/44(65.9%)	-	-	ASA (1/2/3/4):0/23/9/0	ASA (1/2/3/4): 0/25/19/0	-	-
Randomized Clinical Trials										
S. Liu et al., 2017 [17]	34.9± 11.9	35.4 ± 12.6	41/52 (78%)	38/51 (74%)	22.5± 3.7	22.1 ± 3.8	-	-	-	-
Parapiboon W. et al., 2017 [16]	42.7 ± 12.4	43.8 ± 14.1	24/37 (65%)	27/37(73%)	-	-	Diabetes: 9/37(24.3%)	Diabetes: 6/37 (16.2%)	9/37 (24.3%)	6/37 (16.2%)

**Table 4:** Recipients’ background (B).

Authors, Year	Hemodialysis		Time in Dialysis		Previous Renal Transplant		Deceased Donors		Cold Ischemia Time		Overall Ischemia Time	
	ERAS	Standard Care	ERAS	Standard Care	ERAS	Standard Care	ERAS	Standard Care	ERAS	Standard Care	ERAS	Standard Care
Non-Randomized Studies												
Bruintjes, M. H. D. et al., 2019 [8]	28/40 (70.0%)	20 (50.0%)	-	-	-	-	0/40 (0%)	0/40 (0%)	-	-	2.5 ± 0.41	2.5 ± 0.54
Dias, B. H. et al., 2019 [9]	-	-	18.8± 7.2	19.2± 6.5	12/100 (12%)	13/100 (13%)	71/100 (71%)	78(78%)	-	-	4.7 ± 2.5	3.7 ± 0.5
Kevin A Espino et al., 2018 [10]	-	-	-	-	20/139 (14.4%)	13/95 (13.7%)	125/139 (89.9%)	84/95 (88.4%)	-	-	-	-
Halawa, A. et al., 2018 [11]	-	-	-	-	-	-	75/135 (55.55%)	66/155 (43.7%)	-	-	-	-
Fockens M et al., 2016 [12]	-	-	-	-	22/236 (9.3%)	35/183 (13.7%)	236/236 (100%)	183/183 (100%)	17.5 ± 6.2	17.7 ± 6.4	-	-
Simforoosh N et al., 2016 [13]	-	-	-	-	-	-	24/100 (24%)	33/100 (33%)	-	-	-	-
Huang L., et al. 2012 [14]	172/179 (96.1%)	175/186 (94.1%)	24.8 ± 5.6	25.7 ± 4.8	-	-	179/179 (100%)	186/186 (100%)	-	-	-	-
Swarzbach M. et al., 2010 [15]	-	-	-	-	-	-	29/32 (90.6%)	36/44 (81.8%)	14.7	13.6	-	-
Randomized Clinical Trials												
S. Liu et al., 2017 [17]	-	-	-	-	-	-	0/52 (0%)	0/51(0%)	-	-	2.3 ± 0.6	2.4 ± 0.72
Parapiboon W. et al., 2017 [16]	36/37 (97.3%)	31/37 (83.2%)	45	24	-	-	15/37(40.5%)	16/37 (43.3%)	-	-	-	-

**Table 5:** The different synergistic ERAS interventions presented in the included studies are summarized. (A) ERAS Interventions: preoperatively; (B) ERAS Interventions: intraoperatively; (C) ERAS Interventions: post-operatively.

**A. ERAS Interventions: preoperatively**
1. Outpatient workup: body weight optimization, blood pressure control, spirometry and smoking cessation.
2. Outpatient consultation for provision of information on the ERAS protocol, for managing expectations with regards to length of hospital stay and for obtaining informed consent.
3. Carbohydrate loading in non-diabetic patients. Less than 4 h fasting, preoperatively. Avoid maintenance fluids in these patients during the immediate preoperative period.
4. Antibiotic Prophylaxis.
5. Application of Thrombo-Embolus Deterrent (TED) Stockings.
**B. ERAS Interventions: intraoperatively**
1. Urinary catheter placement.
2. JJ stent placement with an intention for early removal postoperatively. The stent can be attached to the tip of the catheter.
3. Surgical Drain placement in the retroperitoneal space/transplanted iliac
4. Wound infiltration catheter placement for continuous administration of local anesthetic. Alternatively, long-lasting local anesthetic injection in the subfascial plane.
5. Goal Directed Fluid Therapy with non-invasive cardiac output monitoring (transesophageal doppler) throughout the procedure is preferable. Target MAP of 75 mmHg. When the need for inotropes or thymocyte globulin induction is anticipated, a central venous catheter can be inserted.
6. Opioid + non-opioid intraoperative analgesia
7. Immunosuppression induction when necessary (basiliximab/thymocyte globulin)
**C. ERAS Interventions: postoperatively**
1. Continue immunosuppression induction if necessary (basiliximab/ thymocyte globulin). Early initiation of tacrolimus/cyclosporine on post-operative day 1 (POD1). Target levels standardized for all patients. Use also of MMF/myfortic ± corticosteroid taper for maintenance.
2. UTI antibiotic prophylaxis.
3. Thromboprophylaxis with compression stockings and enoxaparin.
4. Post-operative analgesia with paracetamol, morphine/fentanyl PCA (early wean before POD2) and local anesthetic infusion through the wound infiltration catheter (removal on POD2). When fluids are tolerated, convert IV analgesics to oral.
5. Start sips of water and ice chips/chewing gum on POD0. Minimal IV fluids on POD0, stop IV fluids on POD1. Start liquid diet on POD1, build up to solid diet. Daily laxatives. Castor oil on POD1.
6. Start early mobilization on POD1 along with respiratory exercises. Gradually advance mobilization.
7. Surgical Drain removal on POD2 (if daily drain output ≤ 50 mls)
8. Urinary Catheter Removal on POD2 (only if JJ stent is not attached to the catheter, otherwise catheter remains)
9. POD4 is the target day for hospital discharge. Conditions that should be met: (i) clinical parameters within normal limits (no tachycardia, no temperature, no tachypnoea, no desaturation, no hypo/hypertension); (ii) adequate mobilization; (iii) solid diet tolerated, post-op ileus resolved; (iv) pain controlled with oral analgesics; (v) adequate education over the use of immunosuppressive drugs; (vi) adequate home support
10. Telephone number for consultation available 24/7. If necessary, on-site post-kidney transplant dialysis for 3–4 weeks and use of walk-in infusion centers. Post-discharge outpatient review with surgeons and physicians in 24 h with subsequent visits tailored to patient needs
11. Outpatient early JJ stent removal between POD7 and POD21: either with simple removal of the urinary catheter (if stent and catheter are attached) or with flexible cystoscopy (if catheter has been removed and JJ stent was not attached)

**Table 6:** Post-operative complications, LOS, readmissions, one-year graft and patient survival.

Authors, Year	Overall Complications		Clavien-Dindo I and II Complications		Clavien-Dindo III-V Complications		Recipients’ LOS		Recipients’ Readmissions		Graft Survival up to 1-Year Post Op		Recipients’ one-Year Survival	
	ERAS	Standard Care	ERAS	Standard Care	ERAS	ERAS	ERAS	Standard Care	ERAS	Standard Care	ERAS	Standard Care	ERAS	Standard Care
Non-Randomized Studies														
Bruintjes, M. H. D. et al., 2019 [8]	22/40 (55%)	28/40 (70%)	16/40 (40%)	21/40 (52.5%)	6/40 (15%)	7/40 (17.5%)	6.20 ± 1.56	7.95 ± 2.12	-	-	-	-	-	-
Dias, B. H. et al., 2019 [9]	12/100 (12%)	11/100 (11%)	4/100 (4%)	5/100 (5%)	8/100 (8%)	6/100 (6%)	5 (3–16)	7 (5–14)	11/100 (11%)	9/100 (9%)	96/98 (98%)	94/97 (97%)	-	-
Kevin A Espino et al., 2018 [10]	-	-	-	-	9/139 (6.5%)	2/95 (2.1%)	4.59 ± 0.76	5.65 ± 0.9	38/139 (27.3%)	26/95 (27.4%)	-	-	138/139 (99.29%)	95/95 (100%)
Halawa, A. et al., 2018 [11]	-	-	-	-	-	-	5 [3,4,5,6,7,8,9,10,11,12]	-	7/135 (5.1%)	-	-	-	-	-
Fockens M et al., 2016 [12]	-	-	-	-	-	-	15.0 ± 11.8	14.9 ± 8.8	37/236 (15.7%)	23/183 (12.6%)	220/236 (93.2%)	174/183 (95.1%)	-	-
Simforoosh N et al., 2016 [13]	-	-	-	-	-	-	-	-	-	-	-	-	-	-
Huang L., et al. 2012 [14]	-	-	-	-	-	-	-	-	-	-	-	-	179/179 (100%)	186/186 (100%)
Swarzbach M. et al., 2010 [15]	10/32 (31.3%)	12/44 (27.3%)	9/32 (28.1%)	9/32 (28.1%)	1/32 (3.1%)	1/32 (3.1%)	18.3 ± 3.1	21.4 ± 3.2	-	-	30/32 (93%)	41/44 (93%)	31/32 (96.9%)	43/44 (97.7%)
Randomized Clinical Trials														
S. Liu et al., 2017 [17]	-	-	-	-	-	-	-	-	-	-	-	-	52/52 (100%)	51/51 (100%)
Parapiboon W. et al., 2017 [16]	-	-	-	-	-	-	-	-	-	-	-	-	37/37 (100%)	37/37 (100%)

**Table 7:** Recipients’ urological complications.

Authors, Year	Urological Complications		Urinary Leakage		Ureteral Stenosis/Obstruction		UTI	
	ERAS	Standard Care	ERAS	Standard Care	ERAS	Standard Care	ERAS	Standard Care
Non-Randomized Studies								
Bruintjes, M. H. D. et al., 2019 [8]	7/40 (17.5%)	7/40 (17.5%)	1/40 (2.5%)	2/40 (5%)	1/40 (2.5%)	3/40 (7.5%)	2/40 (5%)	0/40 (0%)
Dias, B. H. et al., 2019 [9]	-	-	-	-	-	-	-	-
Kevin A Espino et al., 2018 [10]	4/139 (2.9%)	4/95 (4.2%)	2/139 (1.4%)	2/95 (2.1%)	-	-	-	-
Halawa, A. et al., 2018 [11]	-	-	-	-	-	-	-	-
Fockens M et al., 2016 [12]	106/236 (44.9%)	92/183 (50.3%)	2/236 (0.9%)	3/183 (1.6%)	7/236 (3%)	15/183 (8.2%)	97/236 (41.1%)	75/183 (45%)
Simforoosh N et al., 2016 [13]	-	-	3/100 (3%)	4/100 (4%)	1/100 (1%)	2/100 (2%)	20/100 (20%)	29/100 (29%)
Huang L., et al. 2012 [14]	6/179 (3.3%)	17/186 (9.1%)	2/179 (1.1%)	2/186 (1.1%)	0/179 (0%)	0/186 (0%)	4/179 (2.2%)	15/186 (8.1%)
Swarzbach M. et al., 2010 [15]	-	-	-	-	-	-	-	-
Randomized Clinical Trials								
S. Liu et al., 2017 [17]	3/52 (5.8%)	15/51 (29.4%)	0/52 (0%)	0/51 (0%)	0/52 (0%)	0/51 (0%)	3/52 (5.8%)	15/51 (29.4%)
Parapiboon W. et al., 2017 [16]	19/37 (51.3%)	29/37 (78.4%)	4/37 (10.8%)	2/37 (5.4%)	0/37 (0%)	0/37 (0%)	15/37 (40.5%)	27/37 (72.9%)

**Table 8:** Recipients’ non-urological complications *.

Authors, Year	Symptomatic Lymphocele		Delayed Graft Function		Acute Rejection		Gastroenterological Complications		Infection, Other than UTI	
	ERAS	Standard Care	ERAS	Standard Care	ERAS	Standard Care	ERAS	Standard Care	ERAS	Standard Care
Non-Randomized Studies										
Bruintjes, M. H. D. et al., 2019 [8]	5/40 (12.5%)	1/40 (2.5%)	0/40 (0%)	1/40 (2.5%)	2/40 (5%)	1/40 (2.5%)	4/40 (10%)	12/40 (30%)	2/40 (5%)	7/40 (17.5%)
Dias, B. H. et al., 2019 [9]	-	-	31/100 (31%)	36/100 (36%)	-	-	-	-	-	-
Kevin A Espino et al., 2018 [10]	-	-	58/139 (46.4%)	21/95 (25%)	-	-	51/135 (37.7%)	19/95 (20%)	-	-
Fockens M et al., 2016 [12]	8/236 (3.4%)	8/183 (4.4%)	142/236 (60.2%)	83/183 (45.4%)	31/236 (13.1%)	36/183 (19.7%)	-	-	-	-
Simforoosh N et al., 2016 [13]	-	-	-	-	2/100 (2%)	1/100 (1%)	-	-	1/100 (1%)	2/100 (2%)
Swarzbach M. et al., 2010 [15]	-	-	2/32 (6.3%)	10/44 (22.7%)	0/32 (0%)	5/44 (11.4%)	-	-	-	-

* Studies not reporting recipients’ non-urological complications were excluded from this table.

## Data Availability

The data presented in this study are available on request from the corresponding author. The data are not publicly available in a given respiratory due to logistical limitations (size and complexity of datasets).
